# Treatment and outcome prognosis of patients with high-energy transsyndesmotic ankle fracture dislocation—the “Logsplitter” injury

**DOI:** 10.1186/s13018-016-0502-y

**Published:** 2017-01-10

**Authors:** Zhe Wang, Xin Tang, Shenglong Li, Xiuhui Wang, Liangfeng Gong, Tao Zhong, Kunzheng Wang

**Affiliations:** 1 Department of Orthopedic Trauma, The First Affiliated Hospital of Dalian Medical University, Dalian, Liaoning 116011 China; 2 Department of Orthopedics, The Second Affiliated Hospital of Xi’an Jiaotong Univeristy, No.157, Rd Xiwu, Xincheng District, Xi’an, Shaanxi Province 710004 China; 3 Department of Orthopedics, Shanghai Zhoupu Hospital of Pudong New Area, Shanghai, 201318 People’s Republic of China; 4 Department of Bone and Soft Tissue Tumor, Liaoning Cancer Hospital & Institute, Shenyang, Liaoning 110042 China; 5 Department of Orthopedics, Dalian No.3 People’s Hospital, Dalian, Liaoning 116033 China

**Keywords:** Logsplitter injury, Ankle fracture, Distal tibiofibular syndesmotic disruption, Syndesmosis

## Abstract

**Background:**

This study aimed to retrospectively review the clinical efficacy of open reduction and internal fixation (ORIF) for treatment of high-energy transsyndesmotic ankle fracture dislocation—the “Logsplitter” injury.

**Methods:**

Between December 2006 and December 2014, 41 patients (29 males and 12 females; mean age, 41.46 ± 13.42 years) with Logsplitter injury were treated by ORIF procedure. Patients were grouped as typical injury (mainly vertical axial stress) and untypical injury (mainly rotational stress) according to the injury mechanism and the degree of the talus wedged into the distal tibiofibular joint.

**Results:**

After the follow-up of 32.48 ± 24.18 weeks, average American Orthopedic Foot and Ankle Society (AOFAS) score at final follow-up was 78.54 ± 10.66 and the excellent and good rate of 82.9%. Three patients in typical group developed nonunion, and other three cases had infection vs. none in untypical group (both *P* = 0.053). Burwell-Charnely scoring system revealed anatomic reduction of fracture was achieved in 22 cases, fair reduction in 16 cases, and poor in only 3 cases. Patients in untypical group had better fracture reduction (*P* = 0.015) and lower incidence rate of posttraumatic ankle arthritis (*P* = 0.042) than typical cases as well as the range of motion (*P* < 0.01).

**Conclusions:**

The ORIF may be an optimal approach to treat Logsplitter injuries. Patients with untypical injury had better fracture reduction, range of motion, and low incidence rate of posttraumatic ankle arthritis than those typical ones, and the postoperative outcome was affected by the injury and treatment characteristics.

## Highlights

1. Cases of untypical injury had better fracture reduction and range of motion than typical ones.
2. Untypical cases had low incidence rate of posttraumatic ankle arthritis than typical ones.
3. Postoperative outcome was associated with the injury and treatment characteristics.

## Background

**Fig. 1:**
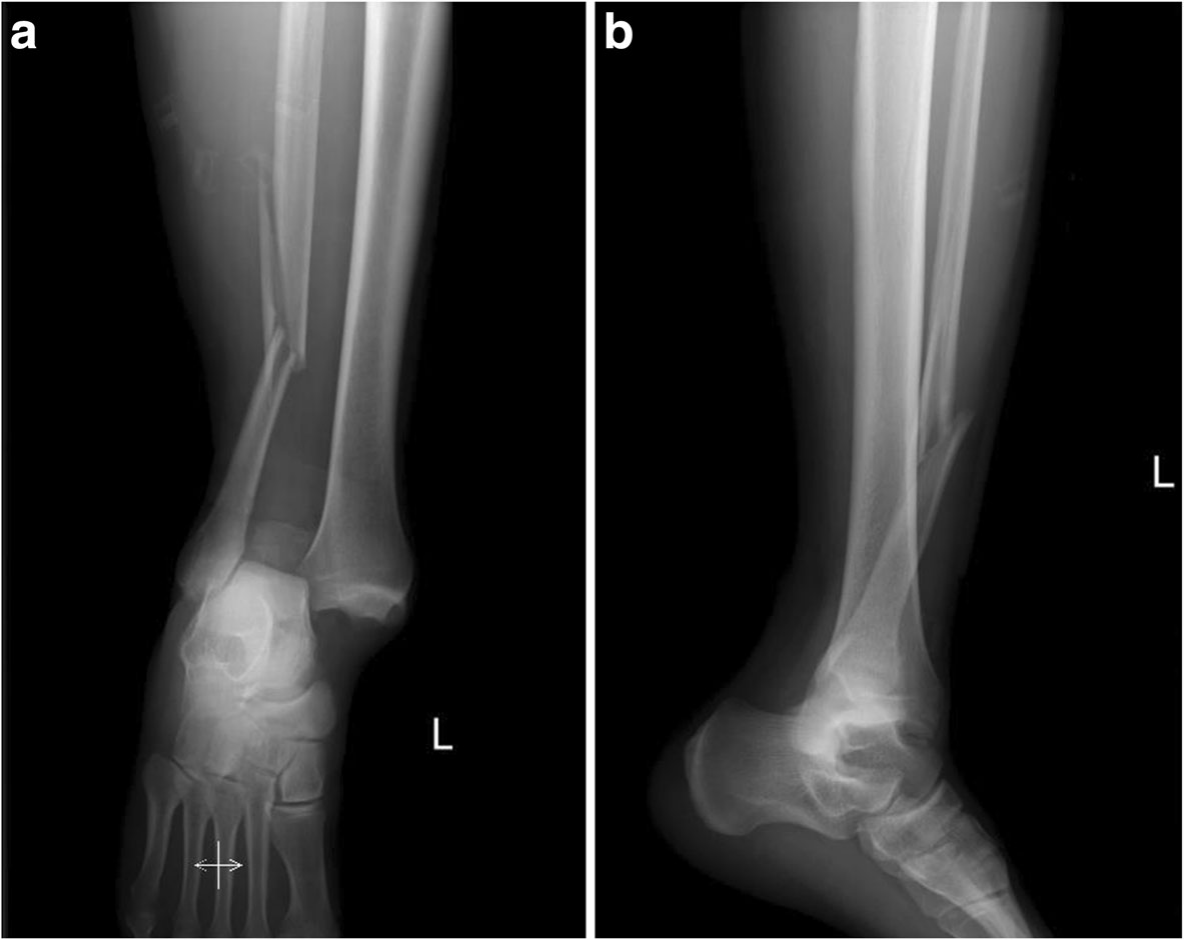
The anteroposterior (**a**) and lateral (**b**) radiographs of typical Logsplitter injury

Ankle fracture is most common among intraarticular fractures of a weight-bearing joint, accounting for 9% of all fractures [1–3]; it mainly included unimalleolar, bimalleolar, and trimalleolar fractures. Distal tibiofibular syndesmotic disruption is a common associated injury with ankle fracture dislocations. It usually results from lower energy rotational mechanisms of injury [4]. Besides, such injury may also occur by way of a high-energy vertical axial violence or a rotational force transmitted to the syndesmotic complex and fibula. However, cases with high-energy transsyndesmotic ankle fracture dislocations, or “Logsplitter” injuries, have been rarely reported, not to mention the treatment. This injury mechanism may be described as similar (although inverted) to a logsplitter wedge, a piece of equipment used with a sledgehammer to split the firewood [4]. Patients with Logsplitter injuries typically present with ankle fracture along with the talus wedged into the distal tibiofibular joint (Fig. 1), resulting in a syndesmotic displacement. Besides, Logsplitter injuries may be associated with tibial plafond fractures or surrounding soft tissue compromise [4].

Ankle fracture is usually treated by open reduction and internal fixation (ORIF), despite the possible occurrence of infection, thromboembolic events, and poor wound healing [5–7]. Bible et al. also previously described the injury pattern and outcomes of Logsplitter injuries [4]; however, the treatment and outcome prognosis was not clear as the injury mechanism is so complicated and the classification is undefined. Hence, this retrospective study was aimed to investigate clinical efficacy of ORIF for patients with typical and untypical Logsplitter injuries, and the outcome prognosis was also evaluated.

## Methods

### Patients

This retrospective study reviewed a total of 41 patients (29 males and 12 females) with Logsplitter injury who were admitted to the First Affiliated Hospital of Dalian Medical University, the Third People’s Hospital of Dalian and Shanghai Pudong New District Zhoupu Hospital from December 2006 to December 2014. The inclusion criteria were (1) age range of 20–70 years; (2) consistent with the injuries mechanism and diagnostic criteria of Logsplitter injury; (3) the fracture types of 44A, 44B, or 44C based on the Association for Osteosynthesis/Orthopaedic Trauma Association (AO/OTA) classification [8]; (4) time to admission within 24 h postfracture; and (5) the implants were not taken out during the evaluation period. The exclusion criteria included (1) patient with neglected fracture, pathological fracture, or who was not tolerant to the surgery due to other systemic diseases; (2) who was accompanied with tibial diaphyseal fracture, calcis, or talus fractures; and (3) who had preexisting osteoarthritis, serious cardiovascular and cerebrovascular diseases, liver or kidney disease, and central nervous system disease. Besides, any female patient in gestational period or in lactation was also excluded.

The Logsplitter injury pattern was defined as an ankle fracture dislocation by a vertical axial violence or combined with a possible rotational force, demonstrating disruption of the syndesmosis with axial displacement of the talus within the distal tibiofibular joint above the level of the tibial plafond [4]. Tibiofibular syndesmotic disruption was diagnosed according to Amendola et al. [9] based on the X-radiograph of normal distal tibiofibular syndesmosis: (1) the proximal and distal tibiofibular space of ≤6 mm at the anteroposterior position or a mortise view; (2) the anteroposterior tibiofibular overlap of >6 mm or greater than 42% of the fibula width; (3) the tibiofibular overlap of >1 mm at the mortise view. Any syndesmosis beyond the above ranges is considered syndesmotic disruption or separation.

All the included patients received anteroposterior and lateral X-rays of the fractured ankle preoperatively, including a mortise view. Radiographs were evaluated by an experienced orthopaedic traumatologist to determine the Danis-Weber of associated fibular fractures [10] and Lauge-Hansen classification [11]. In addition, computed tomography (CT) plain scan with three-dimensional reconstruction and magnetic resonance imaging (MRI) examination were also made for all patients to assess the degree and types of injury. All the procedures were approved by the Ethics Committee of our hospital.

The demographic and clinical data were recorded, including age, gender, the causes of fracture, affected side, patency, polytrauma, AO/OTA classification [8], Denis-Weber classification, preoperative tibiofibular width, and associated injuries. Briefly, their mean age at admission was 41.46 ± 13.42 years (range, 20–67 years). Twenty-five cases suffered right ankle fractures, and 16 cases suffered left ankle fractures. The causes of injury were traffic accident in 11 cases, sprains in 3 cases (such as youth during their sports that involves high-velocity and high-impact movements), falling down in 16 cases, falling from a height in 10 cases, and crashes by heavy object in 1 case, respectively. The fractures were categorized into types 44A (*n* = 3), 44B (*n* = 5), and 44C (*n* = 33) by AO/OTA classification. Total 16 patients had open fractures, among whom there were 6 grade II cases and 10 grade IIIA cases according to Gustilo-Anderson classification [12, 13], and the other 25 cases were closed injuries. In addition, fibula fracture occurred in all but 1 patient, and 29 patients had concomitant injuries to the medial malleolus. According to the injury mechanism and the degree of the talus wedged into the distal tibiofibular joint, patients were grouped as typical injury and untypical injury. In detail, typical injury mainly caused by vertical axial stress is a complete dislocation of talus wedging into syndesmosis complex, leading to a full syndesmotic separation, with or without “Plafond” fracture; as for untypical injury, the avulsion fractures such as “Tillaux-Chaput” or “Wagstaffe” and “Volkmann” fragments are firstly caused by rotational force, then partial talus dislocation by vertical force results in incomplete syndesmostic separation, mortise widened and syndesmostic ligaments intact, or slightly injured.

### Operative procedure

After admission, supporter or plaster external fixation was adopted in case of the aggravation of soft tissue injuries. For patients with closed fractures, manual reduction of tibial astragaloid joint was first performed at emergency, followed by continuous traction of calcaneal tuberosity, and then ORIF procedure was initiated until soft tissue swelling regression, good skin condition, and disappearance of wrinkle sign (about 3–4 days); however, for patients with difficulty in manual reduction of tibial astragaloid joint but with fair soft tissue condition, one-stage ORIF was performed at emergency; as for patients with poor soft tissue condition and swelling, open reduction of tibial astragaloid joint was first performed, then followed by continuous traction of calcaneal tuberosity, and finally two-stage ORIF was initiated until soft tissue swelling regressed and skin condition permitted. Open fractures were all treated by emergency operation. With regard to Gustilo type II injury, ORIF was conducted after wound debridement; for type IIIA-contaminated wound, external fixation using supporter was adopted temporarily after debridement. If patient was accompanied by severe soft tissue loss such as open skin avulsion, the vacuum-sealed dressings (VSD) were used to cover the affected area, and then two-stage surgery was conducted until skin conditions permitted.

The surgery was performed by three fellowship-trained orthopaedic trauma surgeons as the first surgeons in the three hospitals, respectively. Patient was placed in a supine position on a radiolucent operating table. Following general anesthesia or block anesthesia, or continuous epidural anesthesia or continuous epidural anesthesia with intravenous anesthesia, the fracture area was reconfirmed under C-arm fluoroscopy and the surrounding fragments such as medial or posterior malleolus fractures, Volkmann fracture, or Chaput fracture were also determined. A pneumatic tourniquet was placed on the affected limb which was elevated 15 cm. The incision was determined according to the occurrence of medial malleolus fracture manifested in the preoperative radiograph. If medial malleolus fracture was apparent, a medial “J” incision was preferred and extended to the medial malleolus tip. Then, the medial soft tissues were released, and the medial malleolus fragments were temporarily left unfixed, aiming to decrease the reduction pressure of tibial astragaloid joint during the subsequent surgical procedure. Surgical approach (partial anterior or posterior) was dependent on the fragment size of anterior-posterior malleolus and the degree of displacement. After the incision of skin, the subcutaneous tissue was incised by sharp dissection to expose the broken ends of fractured fibula and then the distal reduction of tibial astragaloid joint was conducted and stabilized. Fibular length was firstly restored using a 3.5-mm locking compression plate (LCP). The anterior-posterior malleolus fractures were fixed using lag screws or 3.5-mm plate, and the medial malleolus fracture was fixed using two 4-mm cannulated screws or Kirschner wire via the medial incision. In addition, distal tibiofibular syndesmotic disruption and triangular ligament injury were evaluated intraoperatively by pulling on the fibula in the coronal plane with a bone hook (“Hook” test) or by stabilizing the distal tibia and applying lateral force to the foot (“Cotton” test). Patient with a negative “Hook” or “Cotton” test didn’t need to receive syndesmotic fixation; otherwise, syndesmotic screw fixation was performed by screwing 1–3 3.5-mm syndesmotic screws ectoentad (25–30°) at 3–4 cm above distal tibiofibular syndesmosis. If triangular ligament injury occurred, one-stage repair or reconstruction was conducted. Volkmann fragments were fixed using the plates or the cannulated screws via the lateral approach, whereas Chaput fragments were fixed using the cannulated screws via the medial approach. After good anatomic reduction was achieved under C-arm fluoroscopy, plaster external fixation were adopted to stabilize the fracture if the reduction was understable. Finally, the pneumatic tourniquet was deflated after drainage, and hemostasis was performed to avoid postoperative complication such as hematocele at the wound. Then the wound was closed in layers with 2/0 absorbable sutures in the standard fashion.

### Postoperative management

Patients were advised to take early functional exercise such as the mobilization of toes and knee joint and were forbidden to avoid weight bearing at the affected limb. Dorsal expansion and plantarflexion commenced 2–3 weeks after surgery to avoid ankylosis and traumatic arthritis. Patients were followed up by radioscopy for the wound, bone union, range of motion, ankle joint function, and postoperative complications at postoperative 6 weeks, 3 months, 6 months, and 1 year. Bone union was assessed on the base of Burwell-Charnley radiographic criteria [14]. Functional outcomes were evaluated at the last follow-up by American Orthopedic Foot and Ankle Society (AOFAS) ankle-hindfoot scale (excellent, 90–100; good, 75–89; fair, 50–74; poor, <50) [15–17].

### Statistical analysis

Data were presented as count (percentage) or mean ± standard deviation (SD) and analyzed by independent *t* test, *x* ^2^ test, and one-way analysis of variance using SPSS 19.0 for windows (SPSS Inc., IL, USA). A *P* value of ≤0.05 was regarded as statistically significant.

## Results

In this study, 19 patients and 22 patients were assigned to the typical injury group and untypical injury group according to the injury mechanism and the displacement degree of the talus within the distal tibiofibular joint, respectively. The basic characteristics and surgical treatments of 41 patients with the Logsplitter injury are shown in Tables 1 and 2. In the comparisons between the typical and untypical groups, cause of injury, occurrence of open fracture, and infra-triangular ligament injury, preoperative tibiofibular width, blood loss, and syndesmotic fixation were significantly different (*P* < 0.05 for all). However, other demographic and treatment characteristics were similar between the two groups of patients.

**Table 1:** Patient and injury characteristics

Characteristics	Typical group (*n* = 19)	Untypical group (*n* = 22)	*P* value
Mean age (range), years	42.05 ± 12.74 (21–67)	42.82 ± 14.26 (20–60)	0.858
Male/female	14/5	15/7	0.699
Causes of injury, *n* (%)			0.006
Traffic accidents	7 (36.8)	4 (18.2)	
Sprain	1 (5.3)	2 (9.1)	
Falling down	2 (10.5)	14 (63.6)	
Crashes by heavy object	1 (5.3)	0	
Falling from a height	8 (42.1)	2 (9.1)	
Affected side, *n* (%)			0.097
Left	10 (52.6)	6 (27.3)	
Right	9 (47.4)	16 (72.7)	
Open fracture, *n* (%)	12 (63.1)	4 (18.2)	0.003
II	2 (10.5)	4 (18.2)	
IIIA	10 (52.6)	0	
Polytrauma, *n* (%)	6 (31.6)	2 (9.1)	0.070
AO/OTA typing, *n* (%)			0.745
44A	2 (10.5)	1 (4.6)	
44B	2 (10.5)	3 (13.6)	
44C	15 (79.0)	18 (81.8)	
Lauge-Hansen typing			0.215
Pronation-external rotation (PER)	10	6	
Pronation abduction (PA)	7	14	
Supination-external rotation (SER)	1	2	
Supination-adduction (SA)	1	0	
Denis-Weber classification^a^, *n* (%)			0.247
Type A	1 (5.3)	0	
Type B	4 (21.0)	2 (9.1)	
Type C	14 (73.7)	20 (90.9)	
Preoperative tibiofibular width, mm	17.24 ± 3.30	9.69 ± 2.55	<0.001
Associated injuries, *n* (%)			
Distal tibiofibular syndesmotic injury	19 (100)	10 (45.5)	<0.001
Triangular ligament injury	9 (47.4)	9 (40.9)	0.678
Fibula fracture	18 (94.7)	22 (100)	0.276
Medial malleolus fracture	14 (73.7)	15 (68.2)	0.699
Tibial plafond injury	7 (36.8)	3 (13.6)	0.084

A *P* value of ≤0.05 was regarded as statistically significant

^a^For patients with fibular fractures

**Table 2:** Treatment characteristics

Characteristics	Typical group (*n* = 19)	Untypical group (*n* = 22)	*P* value
Operative time, h	2.28 ± 0.75	1.84 ± 0.75	0.063
Blood loss, ml	121.05 ± 57.72	88.64 ± 41.78	0.044
Syndesmostic fixation, *n* (%)			<0.001
No syndesmotic screw	0	12 (54.5)	
One syndesmotic screw	0	10 (45.5)	
Two syndesmotic screws	17 (89.5)	0	
Three syndesmotic screws	2 (10.5)	0	
Fibula fixation, *n* (%)			
3.5-mm LCP	18 (94.7)	22 (100)	0.276
Medial malleolus fixation, *n* (%)			0.157
Two 4-mm cannulated screws	14 (73.7)	13 (59.1)	
Kirschner wire	0	2 (9.1)	

A *P* value of ≤0.05 was regarded as statistically significant

*LCP* locking compression plate

Intraoperative fluoroscopy showed that 19.5% (8/41) patients had Tillaux-Chaput fragments. Tibial pilon was fractured in 12 (29.3%) of 41 injuries, and the tibial plafond was involved in 10 (24.4%) of total injuries. In addition, there were 34.1(29.3%) cases of Volkmann fracture and 3 (7.3%) cases of Wagstaffe-Le Fort fracture. No obvious Maisonneuve fragments were noted in any of the patients.

**Fig. 2:**
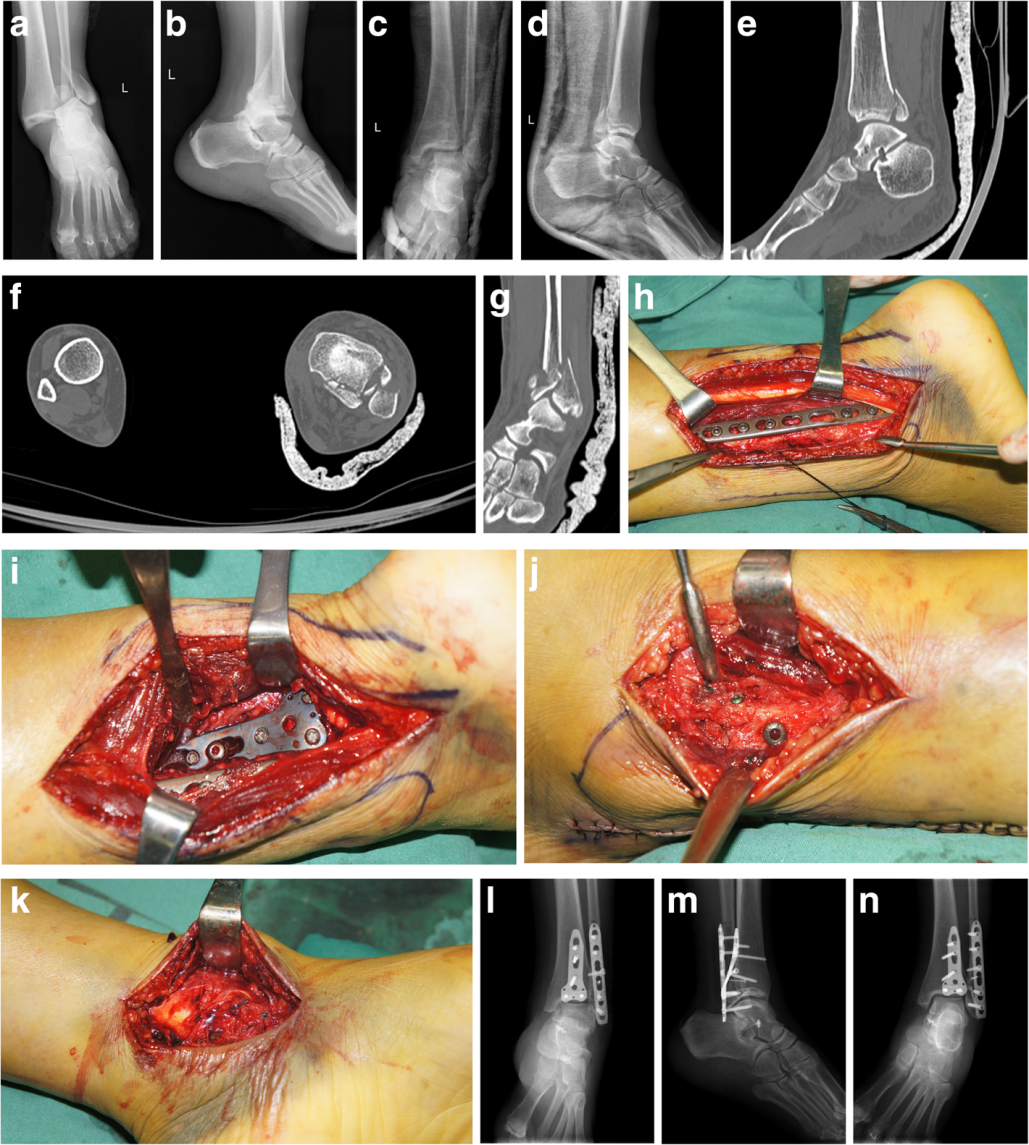
Pre-reduction (**a** anteroposterior; **b** lateral) and postreduction (**c** anteroposterior; **d** lateral) radiographs from a 52-year-old woman after falling down (untypical Logsplitter injury). The CT scans (**e**, **f**, **g**) indicated peroneal Wagstaffe-Le Fort fracture and posterior Volkmann fracture. During the surgery, fibular (**h**) and Volkmann fragments (**i**) were fixed using the plates; Wagstaffe fragments (**j**) were fixed using the cannulated screws; triangular ligament and medial soft tissues were restored (**k**). The anteroposterior (**l**), lateral (**m**), and mortise (**n**) radiographs at the final follow up showed good reduction

**Fig. 3:**
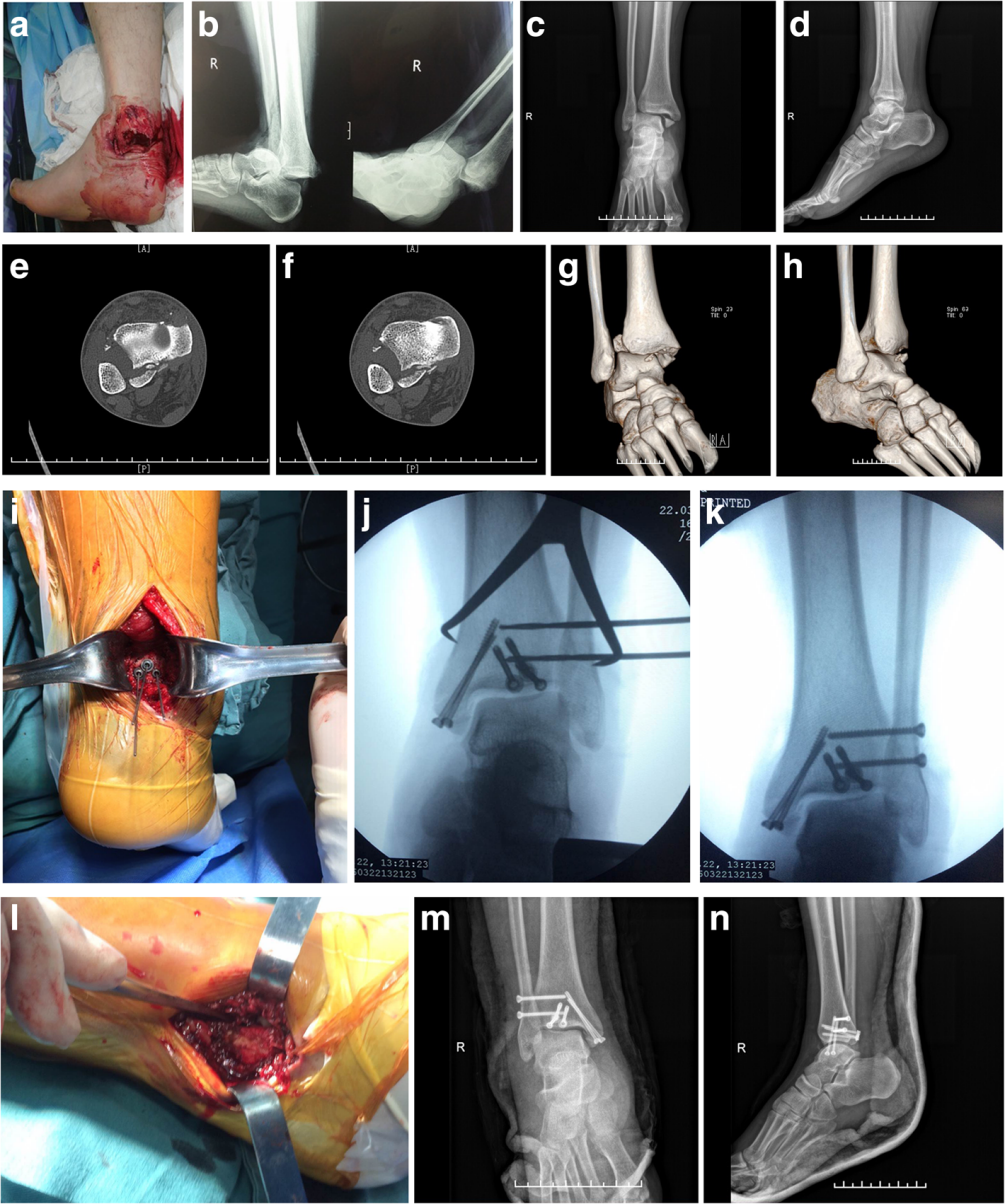
Prereduction (**a**, **b**) and postreduction (**c** anteroposterior; **d** lateral) radiographs from a 44-year-old man after traffic accident (typical Logsplitter injury). The CT plain scan (**e**, **f**) with three-dimensional reconstruction (**g**, **h**) revealed increased tibiofibular width and Volkmann fragments. During the surgery, Volkmann fragments were fixed using the cannulated screws (**i**); syndesmotic screw fixation was performed by screwing 3.5-mm syndesmotic screws (**j**. **k**); medial malleolus fragments were fixed using the cannulated screws (**l**). The anteroposterior (**m**) and lateral (**n**) radiographs at the final follow-up showed satisfactory reduction

All patients were successfully followed up for a mean period of 32.48 ± 24.18 weeks, and the outcome measurements were summarized in Table 3. The preoperative tibiofibular widths were 17.24 ± 3.30 and 9.69 ± 2.55 mm in typical and untypical groups, respectively (*P* < 0.001). After the surgery, tibiofibular width decreased to 5.52 ± 0.66 and 5.60 ± 0.47 mm (*P* = 0.660). The average AOFAS score at final follow-up was 78.54 ± 10.66, with 4 cases rated as excellent, 30 as good, 4 as fair, and only 3 as poor; and the excellent and good rate of 82.9% at final follow-up. Patients between the two groups had similar AOFAS scores at the final follow-up (75.05 ± 13.86 vs. 81.55 ± 5.60, *P* = 0.051). Three of the 19 patients in typical group developed nonunion, and other three cases had infection vs. none of 22 patients in untypical group (both *P* = 0.053). Twenty-eight (68.3%) of 41 patients had radiographic evidence of posttraumatic ankle arthritis, 16 cases in typical group, and 12 in untypical group (*P* = 0.042). According to Burwell-Charnely scoring system, anatomic reduction of fracture was achieved in 22 cases, fair reduction was obtained in 16 cases, and poor reduction in only 3 cases; and the good and fair rate was 92.7%. Patients in untypical group had better fracture reduction than typical cases (*P* = 0.015) as well as the range of motion, including dorsal expansion, plantarflexion, eversion, and inversion compared with typical ones (*P* < 0.01 for all). The typical and untypical cases are shown in Figs. 2 and 3.

**Table 3:** Postoperative outcomes of patients with Logsplitter injury

Outcomes	Typical group (*n* = 19)	Untypical group (*n* = 22)	*P* value
Length of follow-up	29.37 ± 23.94	35.73 ± 24.54	0.408
Postoperative tibiofibular width, mm	5.52 ± 0.66	5.60 ± 0.47	0.660
Infection, *n* (%)	3 (15.8)	0	0.053
Fracture nonunion, *n* (%)	3 (15.8)	0	0.053
Posttraumatic ankle arthritis, *n* (%)	16 (84.2)	12 (54.5)	0.042
Range of motion, °			
Dorsal expansion	23.84 ± 2.11	26.14 ± 2.10	0.001
Plantarflexion	25.58 ± 3.10	30.27 ± 2.33	<0.001
Eversion	22.11 ± 3.62	27.59 ± 2.40	<0.001
Inversion	22.84 ± 3.10	28.32 ± 3.68	<0.001
Burwell-Charnely score, *n* (%)			0.015
Good	6 (50.0)	16 (50.0)	
Fair	10 (14.7)	6 (14.7)	
Poor	3 (2.9)	0 (2.9)	
AOFAS score at final follow-up	75.05 ± 13.86	81.55 ± 5.60	0.051

A *P* value of ≤ 0.05 was regarded as statistically significant

*AOFAS* American Orthopedic Foot and Ankle Society

Furthermore, the influence of the general features of patients and treatments to the postoperative outcomes were also analyzed (as shown in Table 4). The results indicated that the incidence of posttraumatic ankle arthritis significantly associated with causes of injury (*P* = 0.004). Burwell-Charnely score was related to causes of injury and syndesmosis fixation (*P* = 0.001 and *P* = 0.033). Range of motion was affected by several factors, including causes of injury, open fracture, distal tibiofibular syndesmotic injury, preoperative tibiofibular width, and blood loss as well as syndesmosis fixation (*P* < 0.05 for all).

**Table 4:** The association between the injury and treatment characteristics with the postoperative outcomes

Parameters	Posttraumatic ankle arthritis (yes/no), *n*	Range of motion, °				Burwell-Charnely score (good/fair/poor), *n*
		Plantarflexion	Dorsal expansion	Eversion	Inversion	
Causes of injury						
Traffic accident	11/0	27.45 ± 3.08	24.27 ± 3.29	24.82 ± 4.12	25.73 ± 3.71	3/8/0
Sprain	2/1	29.33 ± 6.43	25.00 ± 1.00	22.00 ± 3.46	22.33 ± 2.31	1/2/0
Falling down	9/7	29.50 ± 2.50	25.94 ± 2.11	27.06 ± 2.98	28.38 ± 3.83	13/3/0
Crashes by heavy object	0/1	–	–	–	–	0/0/1
Falling from a height	2/8	26.40 ± 4.30	24.40 ± 1.58	22.70 ± 4.47	23.00 ± 4.29	5/3/2
Open fracture						
II	5/1	29.83 ± 2.79	25.83 ± 2.31	27.5 ± 3.62	27.00 ± 5.65	3/3/0
IIIA	8/2	25.40 ± 3.13	23.30 ± 2.63	21.90 ± 3.73	22.20 ± 2.05	3/6/1
Distal tibiofibular syndesmotic injury						
No	6/6	30.00 ± 2.41	25.75 ± 1.86	28.08 ± 2.54	28.75 ± 4.14	9/3/0
Yes	22/7	27.31 ± 3.71	24.7 ± 2.54	23.79 ± 3.95	24.55 ± 3.90	13/13/3
Preoperative tibiofibular width, mm						
0–11	10/8	30.06 ± 2.04	26.33 ± 2.11	27.11 ± 2.91	27.72 ± 3.82	12/6/0
12–24	18/5	26.57 ± 3.80	24.09 ± 2.13	23.43 ± 4.18	24.26 ± 4.23	10/10/3
Blood loss, ml						
<100	11/9	28.63 ± 3.25	25.26 ± 2.20	26.63 ± 3.85	27.32 ± 3.42	13/4/2
≥100	17/5	27.63 ± 3.85	24.91 ± 2.56	23.68 ± 3.83	24.45 ± 4.73	9/12/1
Syndesmosis fixation						
No syndesmotic screw	6/6	30.00 ± 2.41	25.75 ± 1.86	28.08 ± 2.54	28.75 ± 4.14	9/3/0
One syndesmotic screw	6/4	30.60 ± 2.32	26.60 ± 2.37	27.00 ± 2.21	27.80 ± 3.19	7/3/0
Two syndesmotic screws	15/2	25.58 ± 3.28	23.71 ± 2.08	22.00 ± 3.39	23.06 ± 3.13	5/10/2
Three syndesmotic screws	1/1	25.50 ± 0.71	25.00 ± 2.83	23.00 ± 7.07	21.00 ± 2.83	1/0/1

Continuous variables are presented as mean (SD) and were compared by *t* test or one-way analysis of variance, whereas categorical variables are presented as count (percentage) and were compared by *x* ^2^ test. A *P* value of ≤0.05 was regarded as statistically significant

## Discussion

Unlike other ankle fractures with syndesmotic disruption, Logsplitter injuries commonly occur after high-energy vertical mechanisms of injury or with a combined rotational force. There has been a previous report describing the injury pattern and outcomes of Logsplitter injuries [4]; however, the treatment and outcome prognosis was indefinite. Hence, in this retrospective study, we compared the clinical efficacy of ORIF for patients with typical and untypical Logsplitter injuries, which was divided by the injury mechanism and the degree of the talus wedged into the distal tibiofibular joint. The results showed that patients with untypical injury had better fracture reduction and range of motion and low incidence rate of posttraumatic ankle arthritis than those typical ones. Finally, we also found an association between postoperative outcome and the injury and treatment characteristics.

All the patients included in this study received radioscopy, three-dimensional CT scan, and MRI examination preoperatively. It was found that not all patients with Tillaux-Chaput fragments (5 cases), pilon fracture (8 cases), tibial plafond injury (3 cases), or Volkmann fracture (6 cases) had distal tibiofibular syndesmotic injury, even some cases of syndesmosis were undamaged (data not shown). We speculated that the mechanism of Logsplitter injury could be divided into two categories: mainly vertical axial stress and rotational stress. High-energy vertical axial stress directly causes the talus impacting the articular surface of distal tibia, destroying the distal tibiofibular joint, and wedging into it. This injury was associated with syndesmotic disruption or accompanied with talus or plafond fracture. However, rotational stress was more responsible for the Tillaux-Chaput, Wagstaffe, and Volkmann fragments, talus partially wedging into the distal tibiofibular joint. So patients with rotational force-induced injury might have minor syndesmotic injury or integrated syndesmosis.

In addition, the injury mechanism was also used for patient grouping. The results demonstrated patients with untypical injury had better fracture reduction and range of motion and low incidence rate of posttraumatic ankle arthritis than those with typical ones. Besides, all these outcomes had significant association with cause of injury, as evidenced by the fact that most typical injuries were caused by falling from a height and traffic accidents, whereas more than half of untypical cases were resulted from falling down. Essentially, this could be attributed to the injury mechanism: vertical axial stress directly causes irreversible damage to the articular surface of distal tibia, but rotational stress had less effect on the cartilage; the various degrees of the talus wedged into the distal tibiofibular joint should lead to different severities of tibiofibular membrane injury, and severe membrane injury would affect the blood supply of distal fibula and increase the difficulty in fracture reduction, as well as consequent incidence of posttraumatic ankle arthritis [18]. Furthermore, the result also revealed that the range of motion could be influenced by several other factors, such as open fracture, distal tibiofibular syndesmotic injury, preoperative tibiofibular width, and blood loss as well as syndesmosis fixation.

The AOFAS score for our cohort of transsyndesmotic ankle fracture dislocations was 78.54 ± 10.66 at the last follow-up, a little higher than that of the report by Bible et al. (67.0 ± 26.8) [4]. This might be explained by the inclusion of the untypical cases in our study, as patients in Bible’s report all had syndesmotic disruption. However, patients with Logsplitter injuries have worse outcomes than those cases of common ankle fractures (AOFAS = 84–89) [19, 20]. Nevertheless, ORIF procedure was still a preferred therapeutic method for the treatment of Logsplitter injuries: first, anatomic reduction could be achieved as well as a stable internal fixation; second, the fragments and injured soft tissues could be clear in time to decrease the incidence of traumatic arthritis; third, the injured surrounding soft tissue such as ligaments could also be restored; fourth, the time for external fixation could be reduced, making for early functional exercises.

Several limitations in our study must be addressed. First, as the study was not prospective and randomized control trial, comparison between Logsplitter injuries and other ankle fractures with syndesmotic disruption by low-energy rotational mechanisms was not included. Second, the number of cases included in this study and the follow-up period was insufficient. Hence, further study was needed to prospectively collect rather large sample of transsyndesmotic ankle fracture dislocations from multiple research centers and to compare the treatment outcomes of ORIF procedure as well as outcome prognosis.

## Conclusions

The ORIF procedure may be an optimal approach to treat Logsplitter injuries. Patients with untypical injury had better fracture reduction and range of motion and low incidence rate of posttraumatic ankle arthritis than those typical ones. The postoperative outcome was affected by the injury and treatment characteristics. This study provided the surgeon with therapeutic method and prognostic information when counseling patients and their families with similar injuries.

## Abbreviations

AO/OTA: Osteosynthesis/Orthopaedic Trauma Association
AOFAS: American Orthopedic Foot and Ankle Society
LCP: Locking compression plate
MRI: Magnetic resonance imaging
ORIF: Open reduction and internal fixation
SD: Standard deviation
VSD: Vacuum-sealed dressings
